# P-1665. Factors associated with seasonal Influenza and COVID-19 booster vaccination in a multicentre Irish healthcare worker cohort

**DOI:** 10.1093/ofid/ofaf695.1839

**Published:** 2026-01-11

**Authors:** Liam Townsend, Lisa Domegan, Wenzhou Wang, Sean Brennan, Colm Bergin, Catherine Fleming

**Affiliations:** St James's Hospital, Dublin, Ireland, Dublin, Dublin, Ireland; Health Protection Surveillance Centre, Dublin, Dublin, Ireland; Royal College of Surgeons Ireland, Dublin, Dublin, Ireland; St James's Hospital, Dublin, Ireland, Dublin, Dublin, Ireland; St. James Hospital, Dublin, Dublin, Ireland; Galway University Hospital, Galway, Galway, Ireland

## Abstract

**Background:**

The use of vaccination against both SARS-CoV-2 and Influenza is an important tool in preventing healthcare workers (HCW) ill-health and nosocomial patient infection. However, vaccine uptake is highly variable. We utilise a longitudinal HCW study to describe uptake of seasonal Influenza and COVID-19 booster vaccines, as well as factors associated with receipt of vaccine.

**Figure 1:**
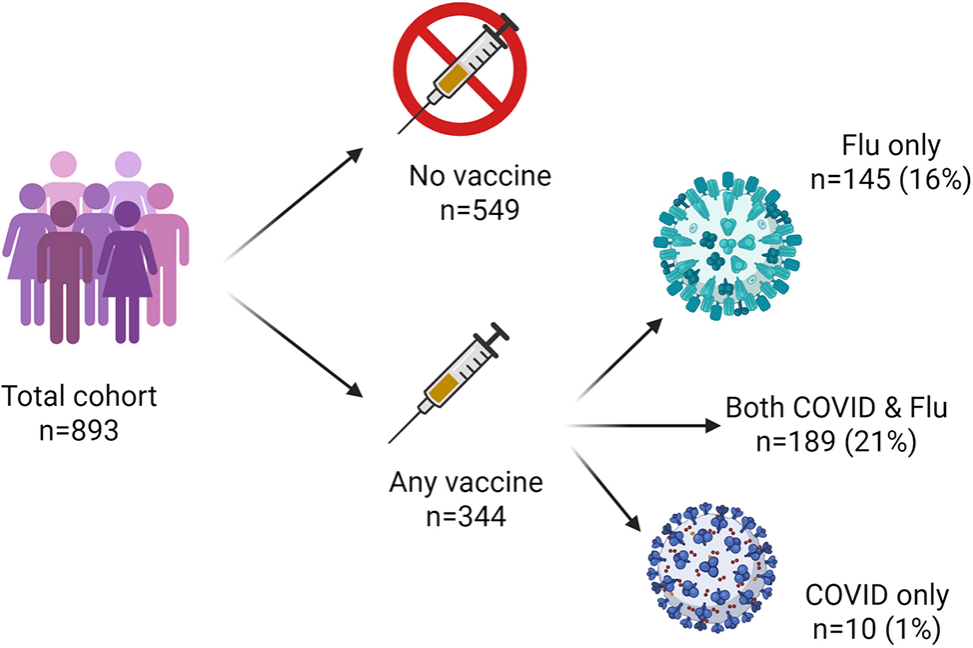
Vaccine uptake Breakdown of Influenza and COVID-19 vaccine uptake

**Table 1:**
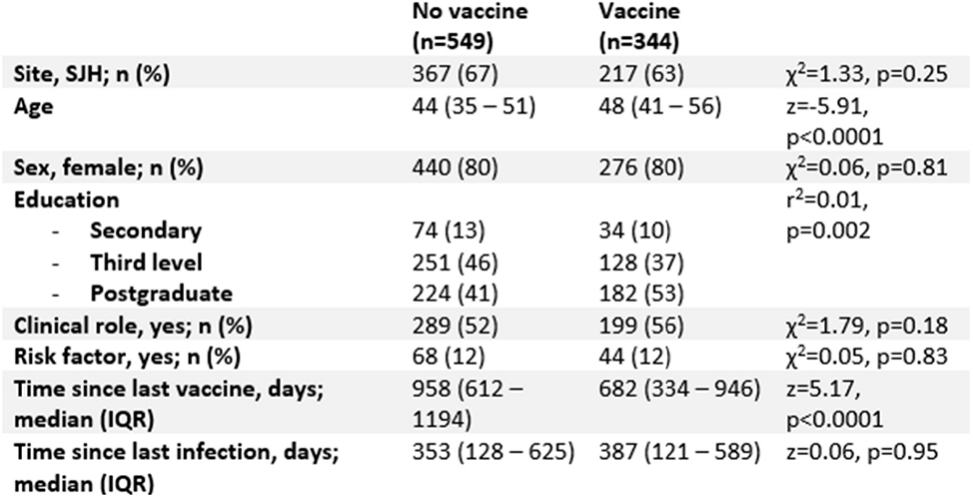
Characteristics associated with receipt of any seasonal vaccine Between-group differences assessed using Chi-squared or Wilcoxon ranksum test, as appropriate.

**Methods:**

All participants in the Prevalence of COVID-19 in Irish HCWs (PRECISE) study, a longitudinal study across two hospital sites in Ireland (St James’s Hospital, Dublin & University Hospital Galway), were included. Participants reported seasonal vaccines received from September 2024 – February 2025, prior vaccine history, and demographic, medical and occupation details. Vaccination history was confirmed using the national immunisation system. Univariate analysis assessed variables associated with both Influenza and COVID-19 vaccine uptake, with multivariable logistic regression including significant univariate variables and relevant variables identified *a priori*.

**Table 2:**
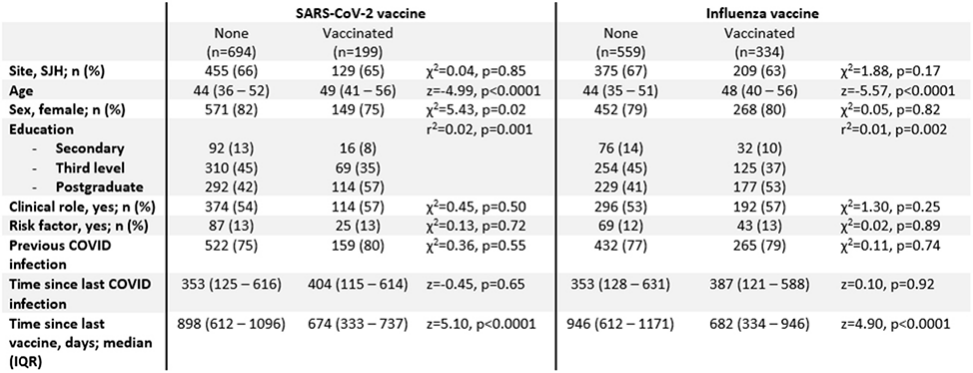
Characteristics associated with COVID-19 booster and seasonal Influenza vaccine Between-group differences assessed using Chi-squared or Wilcoxon ranksum test, as appropriate.

**Table 3:**
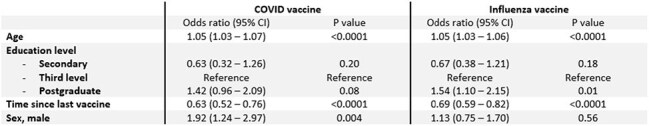
Multivariable regression of factors associated with COVID-19 booster and seasonal Influenza vaccine Multivariable models including significant univariate features and variables selected a priori. Time since last vaccine was z-scored prior to analysis.

**Results:**

There were n=893 participants. Of these, n=549 (61%) did not receive any vaccine, n=189 (21%) received both COVID-19 and Influenza vaccine, n=145 (16%) received Influenza alone and n=10 (1%) received COVID-19 alone (Figure 1). Older age, level of educational attainment, and a shorter interval since most recent vaccination were associated with increased uptake of any vaccine. These variables were associated with both Influenza and COVID-19 vaccine. Additionally, male HCWs were more likely to receive the COVID-booster than females, while there were no sex differences with Influenza vaccine uptake (*Table 2*). These variables remained significant following multivariable logistic regression (*Table 3*).

**Conclusion:**

The overall uptake of vaccination amongst HCWs is low, with 37% receiving Influenza vaccine and fewer than 1 in 4 (22%) receiving a COVID-19 booster. Older age, education status and prior engagement with vaccine programmes influenced vaccine uptake. COVID-19 vaccination was significantly lower in female HCWs. These factors should inform future vaccination information campaigns to improve vaccine coverage.

**Disclosures:**

All Authors: No reported disclosures

